# Serum from Varicose Patients Induces Senescence-Related Dysfunction of Vascular Endothelium Generating Local and Systemic Proinflammatory Conditions

**DOI:** 10.1155/2016/2069290

**Published:** 2016-11-23

**Authors:** Justyna Mikuła-Pietrasik, Paweł Uruski, Krzysztof Aniukiewicz, Patrycja Sosińska, Zbigniew Krasiński, Andrzej Tykarski, Krzysztof Książek

**Affiliations:** ^1^Department of Hypertensiology, Angiology and Internal Medicine, Poznań University of Medical Sciences, Długa 1/2 Str., 61-848 Poznań, Poland; ^2^Department of General and Vascular Surgery, Poznań University of Medical Sciences, Długa 1/2 Str., 61-848 Poznań, Poland; ^3^Department of Pathophysiology, Poznań University of Medical Sciences, Rokietnicka 8 Str., 60-806 Poznań, Poland

## Abstract

Although the role of endothelium in varicose vein development is indisputable, the effect of the pathology on biological properties of endothelial cells remains unclear. Here we examined if the presence of varicose veins affects senescence of endothelial cells (HUVECs) and, if so, what will be the local and systemic outcome of this effect. Experiments showed that HUVECs subjected to serum from varicose patients display improved proliferation, increased expression of senescence marker, SA-*β*-Gal, and increased generation of reactive oxygen species (ROS), as compared with serum from healthy donors. Both increased SA-*β*-Gal activity and ROS release were mediated by TGF-*β*1, the concentration of which in varicose serum was elevated and the activity of which* in vitro* was prevented using specific neutralizing antibody. Senescent HUVECs exposed to varicose serum generated increased amounts of ICAM-1, VCAM-1, P-selectin, uPA, PAI-1, and ET-1. Direct comparison of sera from varicose and healthy donors showed that pathological serum contained increased level of ICAM-1, VCAM-1, P-selectin, uPA, and ET-1. Calendar age of healthy subjects correlated positively with serum uPA and negatively with P-selectin. Age of varicose patients correlated positively with ICAM-1, VCAM-1, and ET-1. Collectively, our findings indicate that the presence of varicose veins causes a senescence-related dysfunction of vascular endothelium, which leads to the development of local and systemic proinflammatory environment.

## 1. Introduction

Varicosity refers to the presence of tortuous, lengthened, and/or twisted veins, typically in the lower limbs superficial or deep inside [1]. In Western nations, the prevalence of varicose veins reaches up to the half of the adult population [2, 3] and rises significantly with age [4, 5]. There is a consensus that varicose veins develop from faulty valves in the veins and weakened vessel walls. Mechanistically, the weakening and a subsequent venous dilatation are evoked by degenerative changes within all layers of the vein wall, in which imbalance between collagen and elastin plays the prominent role [6]. Apart from abnormalities that resulted from the rearrangements in the wall proteins, etiology of varicosity includes hyperproliferation of smooth muscle cells and fibroblasts and an injury-related activation of vascular endothelium [4, 7]. The symptoms and complications of the disease include skin discoloration, pain, itch, ulceration, superficial or deep vein thrombosis, and hemorrhage [8].

Although the role of dysfunctional endothelium in the development of varicose veins is well established [9], the effect of varicosity on endothelial cell biology and, indirectly, on the endothelium-related pathophysiology of varicose vein complications remains elusive. In order to address this issue in a comprehensive manner we compared in this paper serum from patients with varicose veins and from healthy age-matched volunteers in terms of its effect on such critical, functional features of endothelial cells, like proliferation, senescence, production of reactive oxygen species (ROS), and secretory properties. These* in vitro* investigations were followed by a comparative* ex vivo* analysis of both groups of sera with respect to the level of eight arbitrarily selected mediators of vascular inflammation (ICAM-1, VCAM-1, E-selectin, P-selectin, uPA, PAI-1, ET-1, and TFPI) and by studies in which a concentration of these molecules in the sera was correlated with the calendar age of the serum donors.

## 2. Material and Methods

### 2.1. Materials

Unless otherwise stated, all chemicals and culture plastics were purchased from Sigma (St. Louis, MO). Exogenous, recombinant form of human TGF-*β*1 and the specific TGF-*β*1 neutralizing antibody were obtained from R&D Systems (Abingdon, UK).

### 2.2. Varicose Vein Patients and Healthy Donors

The study was performed with serum samples obtained from patients with primary varicose veins of lower extremities and from volunteers (blood donors) in whom the presence of varicose veins was excluded and who were treated as the control group. Varicose patients had symptomatic disease (C_2_E_P_A_S2,3_P_R_) and the inclusion criteria for the study were based on clinical examination. Blood from varicose patients was taken 1 h before planned surgery of varicose vein removal. Blood samples were centrifuged immediately after collection and serum obtained was stored in aliquots at −80°C until required. The study was approved by the institutional ethics committee (consent number 441/13). Demographic data regarding varicose patients and the control individuals are presented in Table 1.

### 2.3. Endothelial Cell Culture and Senescence

Human umbilical vein endothelial cells (HUVECs) were purchased from the American Type Culture Collection (Rockville, MD, USA). The cells were cultured in DMEM with 20% fetal bovine serum (FBS), L-glutamine (2 mM), HEPES (20 mM), EGF (10 *μ*g/mL), heparin (5 U/mL), penicillin (100 U/mL), and streptomycin (100 *μ*g/mL). Senescence of endothelial cells was induced by serial passaging at 7-day intervals until complete exhaustion of cell capacity to divide. Cells from passages 1-2 were treated as “young” cells, while cultures that ceased to divide and displayed hypertrophic appearance were considered as “senescent.” During experiments, young and senescent endothelial cells were plated in culture dishes at high density (80–90% of confluency) and then were simultaneously exposed to 20% serum from varicose vein patients and healthy volunteers for 72 h. Then the cells were carefully washed with phosphate buffered saline (PBS) and exposed for 72 h to serum-free medium to generate autologous conditioned medium.

### 2.4. Immunoassays

Concentration of intercellular adhesion molecule 1 (ICAM-1), vascular cell adhesion molecule 1 (VCAM-1), E-selectin, P-selectin, urokinase plasminogen activator (uPA), plasminogen activator inhibitor 1 (PAI-1), endothelin-1 (ET-1), tissue factor pathway inhibitor (TFPI), growth-related oncogene 1 (GRO-1), transforming growth factor *β*1 (TGF-*β*1), tumor necrosis factor *α* (TNF*α*), and interleukin 6 (IL-6) in serum and/or in conditioned medium generated by endothelial cells was measured using appropriate DuoSet® Immunoassay Development kits (R&D Systems), according to manufacturer's instructions.

### 2.5. Proliferation and Senescence Assays

Endothelial cells were seeded into culture dishes at low-density (5 × 10^4^ cells per well), allowed to attach for 2 h, and then growth synchronized by serum deprivation for next 4 h. Afterwards, the cells were exposed to 20% serum obtained from patients with varicose veins and from healthy volunteers for 72 h. After the incubation, cells were detached enzymatically, counted using the Bürker chamber, and the number of population doublings (the measure of cell proliferation) was calculated using the following formula: population doublings = log_2_⁡(*C*_*t*_/*C*_*o*_), where *C*_*o*_ indicates the number of cells inoculated, and *C*_*t*_ is the number of cells harvested.

The activity of cellular senescence biomarker, senescence-associated *β*-galactosidase (SA-*β*-Gal), was quantified in the same cultures by measuring the rate of conversion of 4-methylumbelliferyl-*β*-D-galactopyranose to 4-methylumbelliferone at pH 6.0, essentially as described in [10].

In some experiments, both parameters were measured upon endothelial cell exposure to exogenous, recombinant form of human TGF-*β*1 used in the concentration corresponding to the cytokine's level in serum from patients with varicose veins. In another group of experiments, SA-*β*-Gal was reanalyzed in the endothelial cells subjected for 72 h to serum from varicose patients preincubated with specific TGF-*β*1 neutralizing antibody (400 ng/mL) [11] for 6 h.

### 2.6. Measurement of Reactive Oxygen Species (ROS)

ROS production was assessed in endothelial cells exposed to 20% serum from patients with varicose veins and from healthy volunteers for 72 h. In brief, 1 × 10^5^ cells were incubated in the presence of 5 *μ*M 2′,7′-dichlorodihydrofluorescein diacetate (H_2_DCFDA) (Molecular Probes, Eugene, USA) for 45 min at 37°C. The fluorescence intensity in cell lysates was monitored in a spectrofluorimeter Victor2 (Perkin-Elmer, Turku, Finland) with excitation at 485 nm and emission at 535 nm. In some experiments, ROS production was examined upon endothelial cell exposure to exogenous, recombinant TGF-*β*1 used in the concentration corresponding to the cytokine's level in serum from patients with varicose veins and in the presence of serum from the varicose patients upon its preincubation with the specific TGF-*β*1 neutralizing antibody (400 ng/mL) for 6 h.

### 2.7. Statistics

Statistical analysis was performed using GraphPad Prism™ v.5.00 software (GraphPad Software, San Diego, USA). The means were compared with Mann-Whitney test or Wilcoxon signed-rank test, when appropriate. Correlations were analyzed using the Spearman test. Results are expressed as means ± SEM. Differences with a *P* value <0.05 were considered to be statistically significant.

## 3. Results

### 3.1. Serum from Varicose Patients Causes Endothelial Cell Dysfunction in the TGF-*β*1-Dependent Mechanism

**Figure 1:**
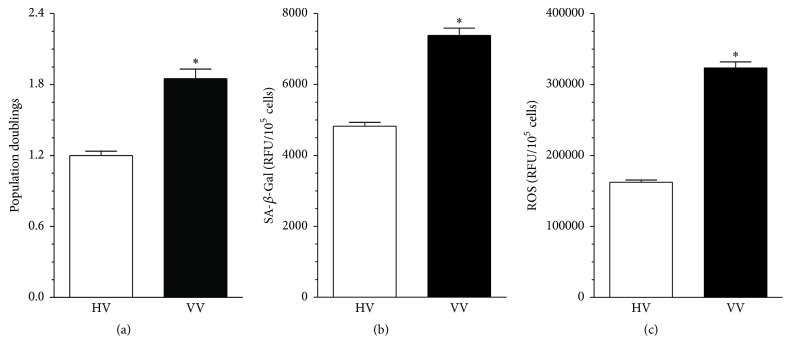
Effect of serum from healthy volunteers (HV) and patients with varicose veins (VV) on proliferation (a), senescence (b), and oxidative stress (c) in cultured endothelial cells. Subconfluent cultures of endothelial cells were exposed to the tested sera (20%) for 72 h and then the number of population doublings achieved (a measure of cell proliferative capacity), the activity of SA-*β*-Gal (a marker of cellular senescence), and the production of ROS (an indicator of oxidative stress) were examined. Results (expressed as means ± SEM) derive from experiments performed with sera from 12 (proliferation) or 16 (senescence, oxidative stress) individuals per group. Asterisks indicate significant differences as compared with HV. RFU: relative fluorescence units.

Low-density cultures of endothelial cells (HUVECs) were exposed to 20% serum from patients with varicose veins (VV) and from healthy, age-matched volunteers (HV) for 72 h and then three functional parameters, that is, cell proliferation, activity of SA-*β*-Gal (biochemical marker of senescence), and production of reactive oxygen species (an indicator of oxidative stress), were examined. Experiments showed that serum from VV patients stimulated proliferation of endothelial cells (the number of population doublings reached increased by 54 ± 8%, *P* < 0.0001; Figure 1(a)), increased the activity of SA-*β*-Gal (by 53 ± 6%, *P* < 0.0001; Figure 1(b)), and increased the level of ROS (by 99 ± 8%, *P* < 0.0001; Figure 1(c)) as compared with the serum from the control donors.

**Figure 2:**
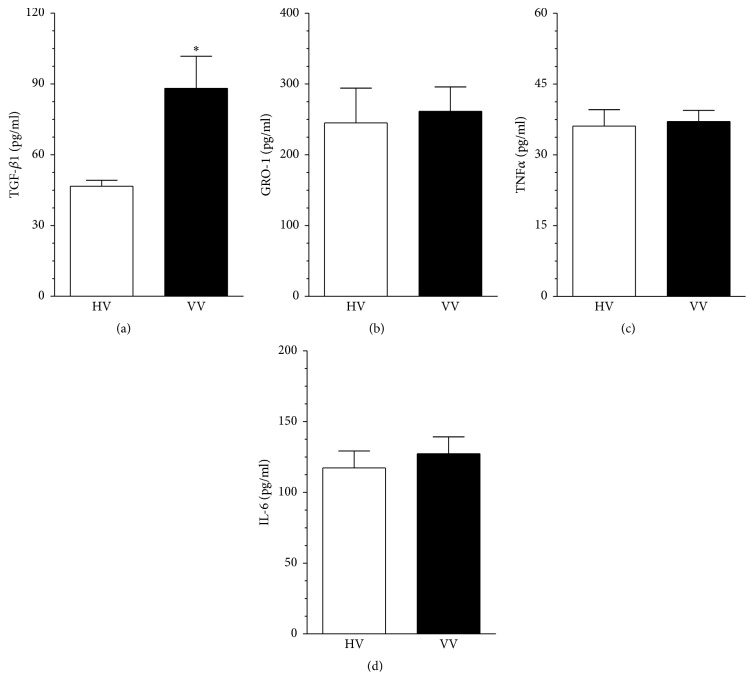
Concentration of TGF-*β*1 (a), GRO-1 (b), TNF*α* (c), and IL-6 (d) in serum from the healthy volunteers (HV) and from the patients with varicose veins (VV). The measurements were made using appropriate ELISA kits. Results (expressed as means ± SEM) derive from experiments performed with sera from 24 individuals per group. Asterisk indicates significant difference as compared with HV.

Intensified proliferation of endothelial cells combined with accelerated senescence and augmented oxidative stress in response to serum from VV patients encouraged us to attempt to identify serum-derived factor(s) which could be responsible for these effects. To this end we selected, based on literature data, four agents (TGF-*β*1, GRO-1, TNF*α*, and IL-6) known to affect cell proliferation [12], senescence [11, 13], and oxidative stress [14, 15]. Comparative analysis of sera from VV patients and healthy volunteers showed that samples differed significantly only with respect to the concentration of TGF-*β*1. Namely, the level of this cytokine in serum from varicose patients was higher (by 87 ± 24%, *P* < 0.003; Figure 2(a)) than in the control group. Concentrations of GRO-1 (Figure 2(b)), TNF*α* (Figure 2(c)), and IL-6 (Figure 2(d)) in both groups of sera were almost identical (*P* > 0.05).

**Figure 3:**
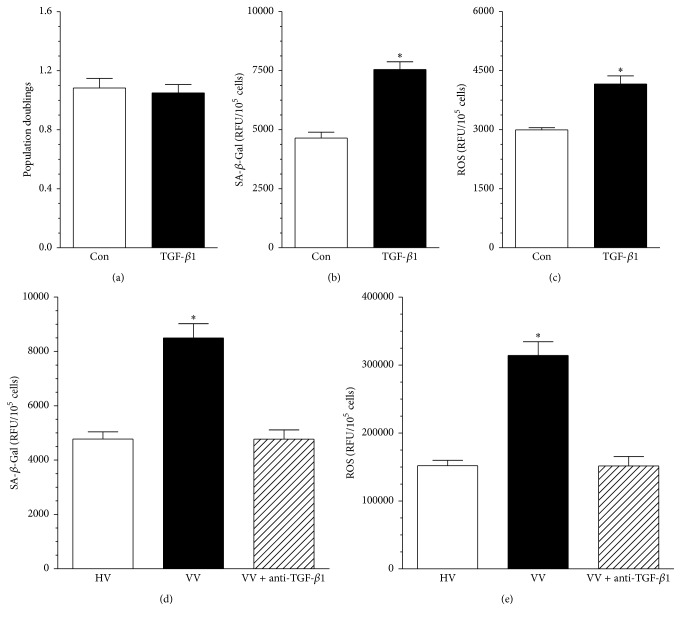
The role of TGF-*β*1 in the dysfunction of endothelial cells subjected to serum from varicose patients (VV). Effect of exogenous, recombinant TGF-*β*1 on proliferation (a), senescence (b), and oxidative stress (c) in HUVECs. The cells were subjected to exogenous protein used at the dose corresponding to its serum (for VV patients) level for 72 h. Asterisks indicate significant differences as compared with the control (Con) group (cells maintained in standard conditions). Effect of TGF-*β*1 neutralization in serum from VV patients on senescence (d) and oxidative stress (e) in HUVECs. Asterisks indicate significant differences as compared with the cells exposed to HV serum. Results (expressed as means ± SEM) derive from experiments performed in octuplicate. RFU: relative fluorescence units.

In order to clarify if the serum from the VV patients exerted its impact on endothelial cells via TGF-*β*1, exogenous recombinant form of this protein was applied to low-density HUVECs at the dose of 100 pg/mL, corresponding to TGF-*β*1 level in serum from varicose patients, and then proliferation, senescence, and oxidative stress were reexamined. This experiment revealed that proliferation of endothelial cells subjected to TGF-*β*1 was unchanged (*P* > 0.05; Figure 3(a)), whereas the activity of SA-*β*-Gal and the production of ROS were increased by 62 ± 9% (*P* < 0.01; Figure 3(b)) and by 39 ± 5% (*P* < 0.04; Figure 3(c)), respectively.

In order to finally confirm the causative role of TGF-*β*1, SA-*β*-Gal and ROS were retested in the endothelial cells subjected to serum from the VV patients in which the cytokine was neutralized using the specific antibody. The study showed that both parameters that were initially elevated in response to serum from VV patients declined to the level characterizing the control group (HV) when the TGF-*β*1 was inactivated (Figures 3(d) and 3(e)).

### 3.2. Serum from Varicose Patients Increases the Secretion of Proinflammatory Agents by Senescent Endothelial Cells

Secretory phenotype (SASP) is one of the most elementary features of senescent cells [16].

**Figure 4:**
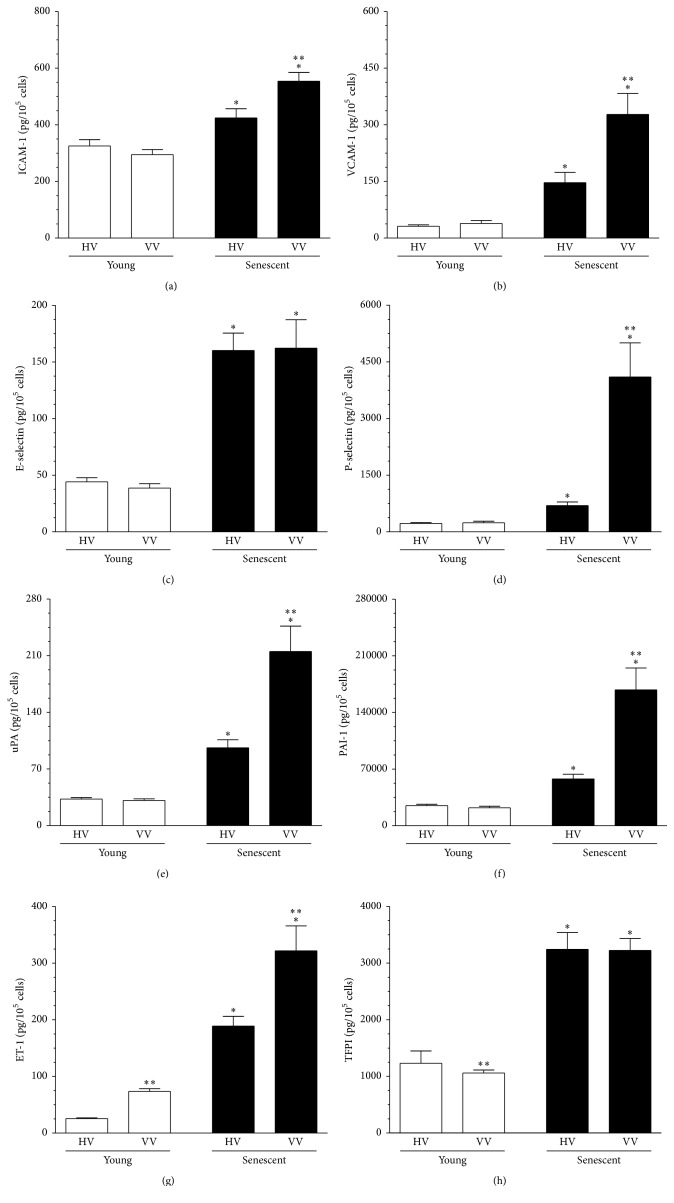
Concentration of proinflammatory agents in conditioned medium generated by young and senescent endothelial cells subjected to serum from the healthy volunteers (HV) and from the patients with varicose veins (VV). Endothelial cells were subjected to 20% serum for 72 h and then they were washed and exposed for next 72 h to serum-free medium to generate conditioned medium in which proinflammatory agents were measured. Results (expressed as means ± SEM) derive from experiments performed with sera from 24 individuals per group. Single asterisks indicate significant differences as compared with young endothelial cells. Double asterisks indicate significant differences as compared with HV.

In this project we induced senescence of endothelial cells by serial passaging and then compared secretory properties of young and senescent cells exposed to sera from varicose patients and from the healthy controls. The study was focused on the release of eight arbitrarily selected mediators of vascular inflammation, that is, ICAM-1, VCAM-1, E-selectin, P-selectin, uPA, PAI-1, ET-1, and TFPI. Experiment showed that senescence of HUVECs maintained in 20% serum from HV displayed upregulated secretion of all tested agents as compared with their young counterparts. The release of ICAM-1 was increased by 30 ± 15% (*P* < 0.01; Figure 4(a)), VCAM-1 by 374 ± 90% (*P* < 0.0001; Figure 4(b)), E-selectin by 263 ± 54% (*P* < 0.0001; Figure 4(c)), P-selectin by 215 ± 69% (*P* < 0.0001; Figure 4(d)), uPA by 191 ± 35% (*P* < 0.001; Figure 4(e)), PAI-1 by 133 ± 27% (*P* < 0.0001; Figure 4(f)), ET-1 by 656 ± 103% (*P* < 0.0001; Figure 4(g)), and TFPI by 163 ± 28 (*P* < 0.0001; Figure 4(h)).

When the reaction of the senescent cells to serum from both groups of donors was compared, it turned out that the conditioned medium generated by cells exposed to serum from VV patients contains remarkably higher concentrations of ICAM-1 (by 31 ± 15%, *P* < 0.02; Figure 4(a)), VCAM-1 (by 122 ± 77%, *P* < 0.01; Figure 4(b)), P-selectin (by 489 ± 208%, *P* < 0.0001; Figure 4(d)), uPA (by 124 ± 59%, *P* < 0.002; Figure 4(e)), PAI-1 (by 190 ± 52; *P* < 0.0001; Figure 4(f)), and ET-1 (by 70 ± 34%, *P* < 0.02; Figure 4(g)). The concentrations of E-selectin (Figure 4(c)) and TFPI (Figure 4(h)), in turn, did not differ (*P* > 0.05) between the groups.

In addition, significant differences were also observed in case of two agents released by young endothelial cells: the concentration of ET-1 in the medium produced by cells exposed to serum from the VV patients was increased by 196 ± 26 (*P* < 0.0001; Figure 4(g)), while the concentration of TFPI produced by these cells was decreased by 14 ± 4% (*P* < 0.01; Figure 4(h)), as compared with cells subjected to serum from HV.

### 3.3. Serum from Varicose Patients Contains Altered Level of Several Proinflammatory Agents

**Figure 5:**
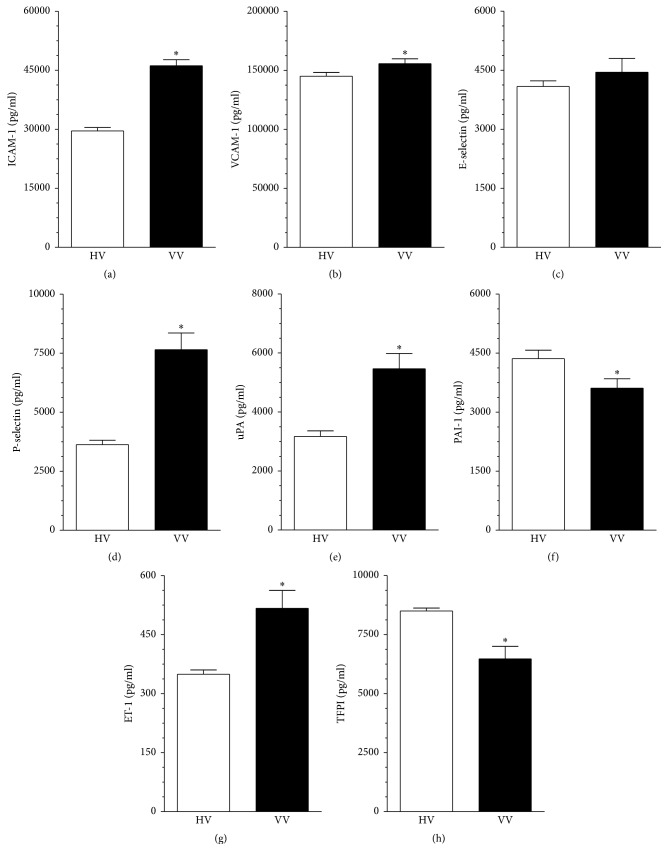
Concentration of proinflammatory agents in serum from the healthy volunteers (HV) and from the patients with varicose veins (VV). The measurements were made using appropriate ELISA kits. Results (expressed as means ± SEM) derive from analysis of sera obtained from 40 individuals per group. Asterisks indicate significant differences as compared with HV.

Concentration of proinflammatory agents tested before in the culture conditions was analyzed again directly in samples of serum from patients with varicose veins and from healthy donors. Study showed that the concentration of five out of eight factors in the serum from VV patients was increased, whereas the concentration of two was decreased and of one remained unchanged. More specifically, the concentration of ICAM-1 increased by 56 ± 8% (*P* < 0.0001; Figure 5(a)), of VCAM-1 by 7 ± 4% (*P* < 0.05; Figure 5(b)), of P-selectin by 111 ± 23% (*P* < 0.0001; Figure 5(d)), of uPA by 72 ± 21% (*P* < 0.0001; Figure 5(e)), and of ET-1 by 48 ± 15 (*P* < 0.001; Figure 5(g)). At the same time, the concentration of PAI-1 decreased by 17 ± 8% (*P* < 0.02; Figure 5(f)) and of TFPI by 24 ± 6% (*P* < 0.0001; Figure 5(h)). The concentration of E-selectin was unchanged (Figure 5(c)).

### 3.4. Varicosity Predisposes to a Positive Correlation between a Serum Level of Proinflammatory Agents and Calendar Aging

**Figure 6:**
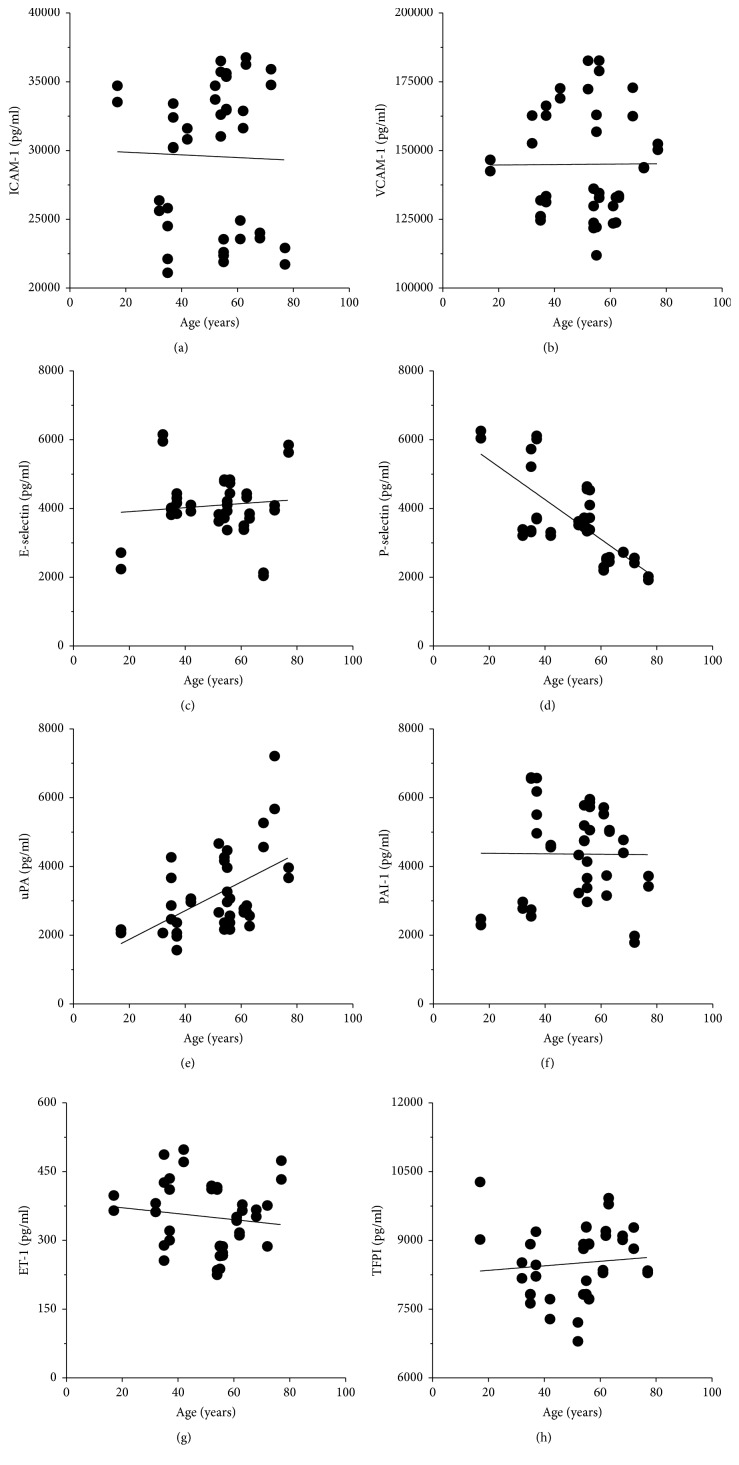
Effect of the healthy volunteers' age on the serum concentration of proinflammatory agents. Correlative analysis was performed with the Spearman test using serum samples from 40 individuals. Exact statistical significance *P* and correlation coefficient *r*-values can be found in Section 3.

It is believed that human aging is associated with a chronic inflammatory response which is manifested by age-dependent increase in the local and systemic concentration of several proinflammatory molecules (so-called* inflamm-aging*) [17]. In this project we aimed to compare an age-dependency of the production of proinflammatory agents in serum from healthy individual and varicose patients. An analysis conducted using control serum from HV showed that aging correlates positively with the concentration of uPA (*P* < 0.02, *r* = 0.4786; Figure 6(e)) and negatively with the level of P-selectin (*P* < 0.0001, *r* = −0.6627; Figure 6(d)). As per the rest of tested agents, there was no relationship (*P* > 0.05) between agents' level and donors' age.

**Figure 7:**
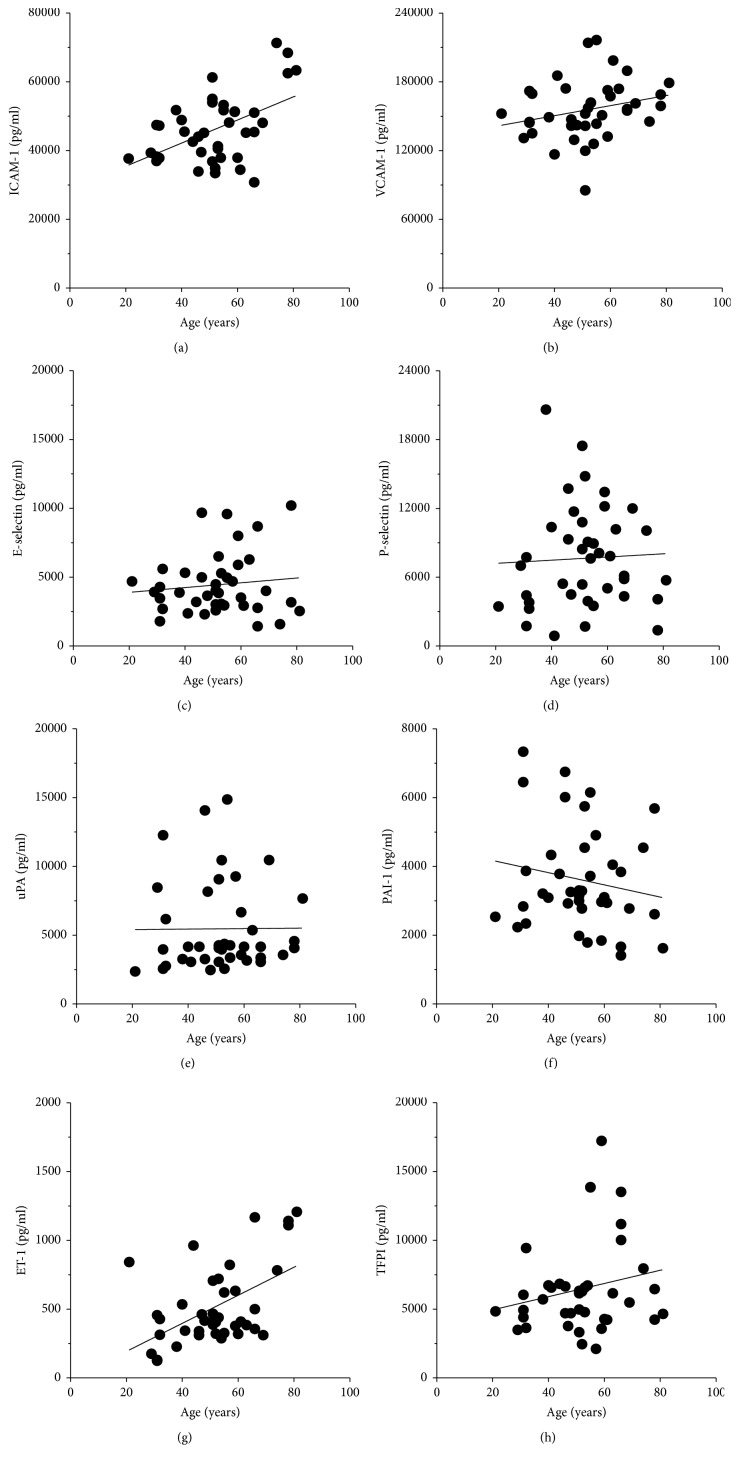
Effect of the varicose patients' age on the serum concentration of proinflammatory agents. Correlative analysis was performed with the Spearman test using serum samples from 40 individuals. Exact statistical significance *P* and correlation coefficient *r*-values can be found in Section 3.

When analogical assessment was performed with sera from VV patients, the results were different. Specifically, aging appeared to correlate positively with the concentration of ICAM-1 (*P* < 0.03, *r* = 0.3581; Figure 7(a)), VCAM-1 (*P* < 0.03, *r* = 0.3550; Figure 7(b)), and ET-1 (*P* < 0.02, *r* = 0.3918; Figure 7(g)). At the same time, there was no relationship (*P* > 0.05) for remaining factors.

## 4. Discussion

Over the past two decades, a body of evidence has accumulated that senescence of cells forming blood vessels (endothelial cells, smooth muscle cells, and fibroblasts) contributes to the development of various vascular pathologies, including atherosclerosis [18, 19], hypertension [20], and impaired healing of venous ulcers [21]. At the same time, the relationship between vascular dysfunction and cellular senescence is clearly bidirectional, which confirmed reports showing accelerated senescence of endothelial cells subjected to atherosclerosis-related disturbances in blood flow [22] or exposed to foam cell-derived lipid peroxidation product, 4-hydroxynonenal [23]. In this paper we described as first that serum obtained from patients with varicose veins causes a senescence-related dysfunction of vascular endothelial cells.

Our considerations on the prosenescence effect of varicose veins stem from the histopathological evaluations by Aunapuu and Arend [24], who found that endothelial cells from varicose patients display specific discontinuity and denudation that may be a manifestation of cellular senescence. In order to verify such a possibility we exposed endothelial cells (HUVECs) to serum from patients with varicose veins and from healthy individuals and found that cells growing in the presence of varicose serum displayed improved proliferative capacity but also increased activity of SA-*β*-Gal, a marker of senescence [25], and increased level of ROS, being the most significant culprits of endothelial cell senescence [26].

Taking into account above-mentioned desquamation of endothelial cells [24], one may assume a scenario that destroyed integrity of cell monolayer in varicose patients may be underlined by oxidative stress-related senescence of these cells, followed by a compensatory intensification of mitotic divisions [27]. Increased expandability of endothelial cells probably reflecting their reaction to the harmful activity of the pathological serum seems to be, however, a blind alley, as it leads to beneficial effect (a restoration of the cell integrity) only temporarily. In long-term perspective, such compensation will result in a premature exhaustion of limited number of achievable divisions, contributing further to the progression of the disease [28]. Such situation has already been described, for example, by Sone and Kagawa, who found that the compensatory proliferation of pancreatic cells driven by insulin resistance resulted in an elevated fraction of senescent cells, which led to even deeper deterioration of insulin activity and diabetes [29].

Comparative analysis of the tested sera as well as intervention experiments with both exogenous protein and specific neutralizing antibody allowed identifying TGF-*β*1 as a plausible mediator by which varicose serum induces senescence and oxidative stress in endothelial cells. This finding is in line with results of Takehara et al., who found that this cytokine is capable of inducing senescence in endothelial cells [12]. Similar effect of TGF-*β*1 has also been found in fibroblasts [30], epithelial cells [31], and mesothelial cells [11]. Moreover, TGF-*β*1 has been recognized to induce ROS in a mitochondria-dependent mechanism [32].

In order to further verify our conception that varicosity potentiates endothelial cell dysfunction in the senescence-related mechanism, we compared directly the reaction of young and senescent HUVECs to sera from both groups of donors. As a measure of endothelium dysfunction we used the cells' ability to hypersecrete mediators of vascular inflammation, including adhesion molecules (ICAM-1, VCAM-1, and E- and P-selectin), vasoconstrictive protein (ET-1), serine protease (uPA), serine protease inhibitor (PAI-1), and the coagulation inhibitor (TFPI). In this context we observed that the release of all tested proteins by senescent cells was remarkably greater as compared with their young counterparts. This finding, confirming the presence of SASP in cultured endothelium is in keeping with reports by other groups, describing, for example, the overproduction of PAI-1 [33], ICAM-1, and VCAM-1 [34] by senescent endothelial cells. Our study enriched, however, the list of factors released at higher amounts to environment by these cells in E-selectin, P-selectin, uPA, and TFPI. Our observations also agree with a general idea of inflammatory phenotype characterizing endothelial cell senescence, depicted at the molecular level in an elegant study by Prattichizzo et al. [35]. More importantly, however, the production of six out of eight tested agents, that is, ICAM-1, VCAM-1, P-selectin, uPA, PAI-1, and ET-1, by senescent cells exposed to serum from varicose patients appeared to be much more pronounced as compared with the control group. Interestingly, further analysis of the concentration of the proinflammatory agents directly in the serum from the varicose patients and from the healthy individuals revealed that the level of five out of six proteins, the secretion of which was upregulated by senescent endothelial cells (apart from PAI-1), is also increased in the first group, which may suggest that senescent endothelial cells may be causatively linked with this phenomenon.

Taking into account the above-mentioned findings one may envisage that pathologies resulting from the presence of varicose veins, in particular thrombotic disorders, may be evoked, at least to some extent, by the proinflammatory behaviour of senescent endothelial cells, efficiently translating to serum characteristics. This argumentation has support in a paper by Tian and Li, who summarized available data to suggest that senescence of endothelial cells may be involved in the development of several vascular pathologies [36]. As per agents tested in our project, the above hypothesis agrees with results of other groups that showed either increased concentration of ICAM-1 and VCAM-1 in serum from varicose patients [37] or the relationship between elevated ICAM-1, VCAM-1, and P-selectin and the development of venous thrombosis [38, 39] and pulmonary embolism [40].

In the last part of the project we examined if the presence of varicose veins may exacerbate a systemic (serum-dependent) phenomenon of inflamm-aging [17]. This hypothesis was based on the facts that (i) serum from varicose patients induces senescence of endothelial cells, (ii) senescent endothelial cells subjected to these sera overproduce proinflammatory agents, and (iii) senescent endothelial cells accumulate in tissues during aging [41]. To clarify this issue we correlated levels of eight proinflammatory agents with calendar age of serum donors. As per the healthy individuals only the concentration of uPA correlated positively with aging (uPA exerts atherosclerotic capabilities [42]), whereas the concentration of P-selectin did correlate negatively. Importantly, however, when the analysis was repeated using the sera from varicose patients, aging appeared to correlate positively with other group of agents, that is, ICAM-1, VCAM-1, and ET-1, whereas the correlations noticed for the healthy donors disappeared. It should be stressed at this moment that the age-dependent decrease in P-selectin should be considered as the beneficial process for healthy people, as this molecule mediates various elements of proinflammatory cascade and contributes to vascular disorders [43]. The lack of the correlation regarding P-selectin in varicose patients, in combination with positive relationships for ICAM-1, VCAM-1, and ET-1 (all are actively involved in atherosclerosis [44, 45]), implies that varicosity makes a serum more proinflammatory, and thus it may be partly responsible for the development of certain age-related pathologies.

## 5. Conclusions

In conclusion, our study provides evidence that serum from patients with varicose veins is capable of generating the proinflammatory local (endothelium-related) and systemic environment. This activity of varicose serum seems to be primarily associated with TGF-*β*1-dependent activation of endothelial cell senescence.

## Figures and Tables

**Table 1:** Characteristics of patients with varicose veins and the control individuals from whom serum samples were taken.

Parameter	Varicose patients	Healthy donors
*n*	40	40
Sex (male/female; *n*)	15/25	17/23
Age (mean ± SD/ range; y)	52 ± 15/21–81	51 ± 15/17–77

	Comorbidities^**∗**^ (number)	

No comorbidities	23	29
Hypertension	6	6
Hypothyroidism	2	0
Asthma	2	0
Obesity	2	2
Psoriasis	3	1
Allergy	1	2
Adrenal insufficiency	1	0

^**∗**^Comorbidities have been treated as follows: hypertension: angiotensin-converting enzyme inhibitors and angiotensin II receptor blockers; hypothyroidism: synthetic analogue of thyroxine; asthma: inhaled sympathomimetic drugs; allergy: antihistamine drugs; adrenal insufficiency: hormone of the adrenal cortex.
